# Piglets Born from Sows Fed High Fibre Diets during Pregnancy Are Less Aggressive Prior to Weaning

**DOI:** 10.1371/journal.pone.0167363

**Published:** 2016-12-01

**Authors:** Thiago Bernardino, Patricia Tatemoto, Beatrice Morrone, Paulo Henrique Mazza Rodrigues, Adroaldo José Zanella

**Affiliations:** 1 Department of Preventive Veterinary Medicine and Animal Health, School of Veterinary Medicine and Animal Science, University of São Paulo, Pirassununga, Brazil; 2 Department of Animal Nutrition and Production, School of Veterinary Medicine and Animal Science, University of São Paulo, Pirassununga, Brazil; University of Lethbridge, CANADA

## Abstract

Aggressive interactions, and their consequences, are the most important causes of poor welfare in piglets. Aggressive behaviour can be modulated by the prenatal and neonatal environment in several species. Commercially kept pregnant sows are often subjected to food restriction, which can compromise their welfare. Limited information is available on the consequences of sow hunger during pregnancy on welfare outcomes for their piglets. High fibre diets can mitigate the feeling of hunger and, consequently, it may improve welfare and productivity measures. The aim of this study was to assess the consequences of feeding pregnant gilts with high fibre diets (HFD) on agonistic behaviour, as manifested by skin lesions, and indicators of fear in their piglets at weaning. Twenty-two pregnant gilts were fed either HFD, 12.86% of crude fibre, 2.4 kg per day (N = 14), or low fibre diet (LFD), 2.53% of crude fibre, 2.0 kg per day (N = 8). During lactation, both treatments received the same diet, *ad libitum*. We investigated the impact of HFD on behaviour and performance measures (birth weight, average daily gain, weaning weight, see S3 File) in the offspring. Skin lesions were evaluated before and after weaning in 156 piglets (100 HFD and 56 LFD), and 142 piglets were subjected to an open field test and a novel object test (87 HFD and 55 LFD). We found no treatment effect on the performance measures. Piglets born from gilts that received HFD had fewer skin lesions before weaning (D28) than the offspring of LFD gilts, while no difference was found during days 29 and 30. In the open field and novel object tests, there was no treatment effect on the behaviour of piglets. The improved skin health at weaning in piglets of sows fed HFD suggests less agonistic interactions amongst these littermates than in piglets of sows fed LFD.

## Introduction

Excessive aggression among pigs is a serious economic and welfare issue [1] and it is also a persistent problem [2]. There are some possible reasons for the occurrence of this behaviour. Prenatal and neonatal environments can modify the behaviour of animals with potential consequences for successive generations [3, 4]. Such characteristics may influence the adaptive response to different challenges [5]. Management strategies focusing on the prenatal and neonatal environments of domestic animals are able to modify immune function [6, 7], fear [8], memory [9, 10] and social behaviour [11], with consequences on the organisation of important coping systems, particularly the central nervous system [12, 13]. There are other factors that may modulate aggressive behaviour, such as the asymmetry in body weight [14], environmental enrichment [15], available space per animal [16] and weaning age. Premature weaning, defined by [17] as weaning before four weeks in pigs, caused memory impairment, altered the expression of stress responsive genes in the brain and increased aggression [9, 10].

An appropriate body condition score of the sows is important in order to avoid obesity and excessive leanness, to prevent low reproductive and performance indices [18, 19, 20]. Therefore, sows are subjected to a feed restriction of approximately 50 to 60% of its *ad libitum* intake capacity or motivation [21]. Food restriction is known as one of the most important sources of stress for pregnant sows [22]. Increase in aggression, occurrence of stereotypic behaviour, reduction in weight gain and changes in food motivation are some of the poor welfare outcomes reported in sows kept on restrictive diets ([18, 23–25, 26] 26-finisher pigs]). Alternative diets using fibre have been the subject of studies to mitigate the negative effects of food restriction and reducing the feeling of hunger, which pregnant sows may experience. The inclusion of fibre in the diet of these animals can reduce the expression of stereotypic behaviour [27, 28], reduce aggression [20], reduce feed motivation [29], increase long-term satiety [30], improve the welfare [31] and improve performance [32]. These cited studies were conducted out in an attempt to understand the effect of a high fibre diet in the behaviour and productivity of the sows themselves. However, as far as we know, no previous study has investigated the effect of a high fibre diet for sows on the behaviour of their piglets.

The skin lesion score is significantly influenced by reciprocal fight and bullying, making this measure an indicator of the outcomes of aggression [33]. The score can be obtained by counting the skin lesions, classified as any scratch found in the skin (old, with scab, or recent lesion) [34]. Behavioural characteristics associated with emotionality, such as fear and anxiety could be important factors to determine the likelihood of an animal to engage in aggressive interactions. Studies using open field and novel object tests may help to identify susceptible and resilient phenotypes regarding their ability to adjust to their social environment, making optimal decisions, and possibly having better welfare outcomes [35]. The emotionality tests can inform about the individual characteristics of animals. This information is crucial to define the strategy chosen by an animal, such as engaging in a fight or not.

The aim of the present study was to investigate the impact of a high fibre diet for pregnant gilts on the levels of skin lesions and behaviour in open field and novel object test in their piglets.

## Material and Methods

### Animals

The experiment was carried in an experimental pig farm at the Campus Fernando Costa, School of Veterinary Medicine and Animal Science, University of São Paulo, Pirassununga, Brazil, upon approval of the Ethics Committee on the Use of Animals (CEUA) at the School of Veterinary Medicine and Animal Science (FMVZ) of the University of São Paulo (USP), under the number 3606300114.

We studied the offspring of 22 sows, distributed by weight, making the groups homogeneous, in high or low fibre diet treatments on the day after they were inseminated with pooled semen. At the beginning of this study, we had 32 sows, 16 in each treatment. However, 7 sows from one and 1 sow from the other treatment returned to oestrus and then, were excluded from analysis, and 2 was excluded for health reasons. The sows were kept in a group-housing system during pregnancy, with individual sows offered either high or low fibre diets during pregnancy, separated during feeding time in closed feeding stalls, in the same pen. Fourteen sows were fed high fibre diet (HFD), (12.86% crude fibre) and eight sows were fed low fibre diet (LFD) (2.53% crude fibre). It was the intention that extra quantities of high fibre diet be sufficient to match the nutrient intake of LFD. LFD animals received 2 kg of diet (3.300 kcal per kg) and HFD animals received 2.4 kg of diet (2.764 kcal per kg) per day, in two equal portions given at 8:00 am and 15:00 pm, 50% in each period.

HFD diets had a 35% inclusion of soybean hulls, and were mixed to the diet. High and low fibre diets were offered throughout the pregnancy. The HFD was composed by 57.6% of corn, 4.9% of soybean meal, 35% of soybean hulls, and 2.5% of vitamin-mineral premix. The LFD was composed by 81.7% of corn, 15.8% of soybean meal and 2.5% of vitamin-mineral premix. Parturition occurred in farrowing pens, where the sows were moved on the 107^th^ day of pregnancy. The lactation diets were the same for both treatments, offered *ad libitum* for all sows, and was composed by 64% of corn, 30% of soybean meal, 3% of soybean oil and 3% of vitamin-mineral premix.

For the analysis of the lesions of the offspring of differentially fed sows, we studied 56 piglets born from LFD sows and 100 piglets born from HFD sows. Lesions were counted in the piglets immediately prior to and during weaning. Measures of emotionality were obtained in the open field and novel object tests, where 142 pigs were investigated, 86 piglets from HFD sows and 56 piglets from LFD sows (see S2 File).

### Facilities and handling

The gestation pens were 6.7 m wide by 4.4 m long, giving 3.3m^2^/sow. Each pen had 9 individual feeding stalls (1.8m x 0.55 m) with a nipple drinker in each stall, with *ad libitum* access to water. The feeders were built of concrete. During feeding time, all animals were confined in the stalls, to avoid intermixture among dietary treatments.

From the 107^th^ day of pregnancy, until the 28^th^ day of lactation, sows were housed in individual farrowing pens measuring 4.3 x 2 m. Connected to the pen, there was a creep area made of concrete (0.97m x 2.2 m), where piglets had unlimited access to solid feed from the first day of life. The sows had access to bedding material composed by dehydrated sugar cane bagasse and hay. The creep area also had bedding material, the same as the pen and a heat source provided by a 60 Watt heat lamp. Farrowing was monitored with IP connected video cameras, with real time internet transmission to the experimenters by computers and smartphones and also through direct observation. Interventions were carried out only when necessary, following a pre-established protocol, enabling standardization of management procedures. The sows were divided in three blocks, according to the gestational period. The first block was composed by 8 animals, the second by 14 and the third by 6 sows, totalizing 28 animals.

One-day old piglets were subjected to a common management protocol: they were weighed, received two hundred milligrams of iron dextran, had their teeth grinded and had their ears notched for identification, using local anaesthesia (cream, lidocaine 50 mg/g). In contrast to common farm protocols, male piglets were not castrated and were not tail docked.

When piglets reached 27 days of age, they were weighed. At 28 days of age they were weaned and allocated to an experimental pen. Piglets were standardized by weight and gender, at least six piglets (three male and three female) from each sow were used to evaluate skin lesions. The criterion of farrowing order was used in the assignment of piglets in weaning pens, as the sows were not subjected to hormonal protocol for oestrus or farrowing synchronization, and housing by uniform body size in the same group. The strategy for animal allocation resulted in four type of pens, "A”, "B", "C" and "D". Pen "A" had the heaviest animals, followed by "B", "C" and "D”, which housed the lightest piglets, in a descending order considering the differences in piglet weight. Each animal was individually identified, using a marker of non-toxic and non-permanent ink. Piglets were allocated either to pens with only one treatment (HFD N = 21 pens or LFD N = 10 pens), each pen having one male and one female piglet each from 2 different litters (N = 4 piglets per pen), or to pens with two treatments mixed (N = 8 pens). For each litter, 2 piglets were allocated to pens for each of the four weight classes, “A”-“D”, totalling an average of 8 piglets per sow. These nursery pens measured 0.75 m^2^ (1 m x 0.75 m) each, with slatted plastic flooring and no sharp edges. Piglets had access to water and food *ad libitum* and the pens were cleaned daily. Weaning, moving piglets to the new pen and the collection of photos and videos were carried out between 7:00 and 7:30 am.

Photos were taken daily to count the lesions, following the methodology of [34] and the novel object and open field tests [35] were held at the end of data collection, three days after weaning.

### Data collection

**Fig 1:**
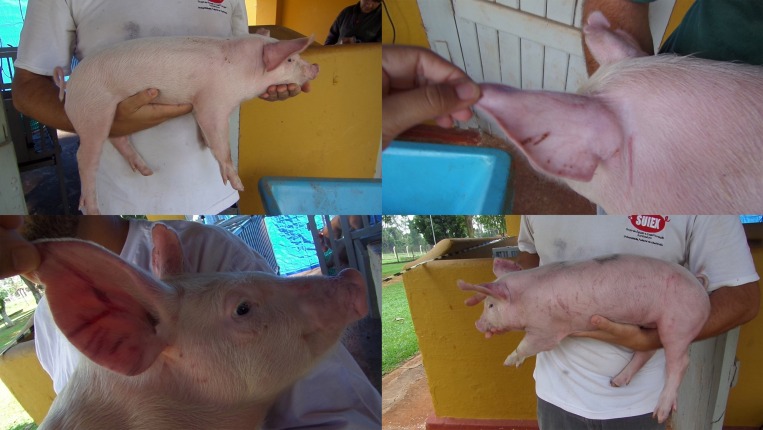
Pictures used to measure the skin lesions

For the counting of lesions, each piglet was photographed and filmed on weaning day (D28, immediately prior to weaning), and during the other two subsequent days, D29 (24 hours after weaning) and D30 (48 hours after weaning). Each piglet was individually restrained and pictures of the body, inner and outer face of the ear, neck and face, on both sides, were captured on 6 photos in total (see Fig 1). The counting methodology used was the total amount of identified lesions in the photos [34]. Two independent evaluators analysed the photos, independently, having no knowledge of treatment allocation for each animal.

The open field test and the novel object test were performed in combination at the end of the D30, and followed similar methodology to that described by [35]. In each weaning group, all tests began approximately at 15:30 pm and lasted for 10 minutes per animal. The pen used in the test had solid concrete floor, measuring 4.85 by 2.37 m; the floor was marked with permanent yellow ink into quadrants of 83 by 78 centimetres. A digital camera was used for video recording. The pen was always washed with water before the start of each test, to minimize interference of olfactory cues. The test order of the piglets was in accordance with the pens, starting with one piglet from pen "A", followed by a piglet from pen "B", "C" and "D", with an interval of 30 minutes between animals of the same pen. The first animals tested in the same pen were littermates of one litter, followed by their sisters. Each animal was placed in the same starting position. All this strategy was performed to standardize at maximum the test handling. Only when all the littermates were subjected to the test, the litter of the other sow, which was in the same pen, was tested. All piglets were tested in the same day.

**Fig 2:**
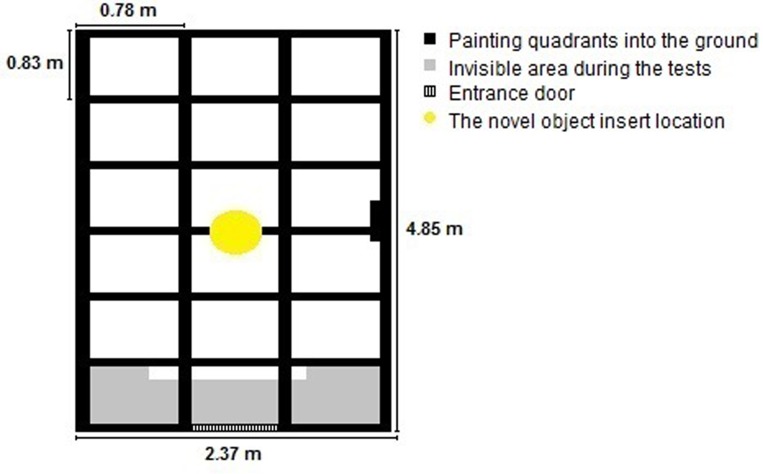
Painting quadrants into the ground used in the open field test and novel object test

The open field test was conducted in the first five minutes and the novel object test in the following five minutes. The object used was a polypropylene bucket with a capacity of 20 litres, empty and yellow. The bucket was suspended and always descended in the centre of the pen, 5 minutes after the start of the open field test, using a pulley mechanism, without visual contact between the animal and the experimenter (Fig 2). Because of the position of the camera, a few quadrants was invisible. However, these quadrants were in the same part of the pen and did not influence on our analysis.

In the open field test, the following measures were taken: piglet’s activity (time spent walking), amount of time the animal remained in the central and lateral quadrants (quadrants on the edge of the pen), latency to move and vocalization (see S2 File). In the novel object test, we measured latency to interact with the object, time of interacting with the object (close to and with the head toward to the object), time the animal remained close to the object (in quadrants that surround the object), and vocalizations (see S2 File). All types of vocalizations was counted for both tests. All quadrants were evaluated in both tests.

The performance data of piglets were obtained during the suckling period. All animals were weighed three times (one day old, 21 days old and 27 days old, see S3 File) and the average daily gain per litter and individual one were calculated according to the piglet´s exact age.

### Statistical analyses

Data were analysed with the package Statistical Analysis System (SAS Inst., Inc., Cary, NC). Initially, the data were analysed for the presence of discrepant information (outliers) and to verify residual normality we used the Shapiro-Wilk test. When the normality assumption was not found, the transformation by logarithmic, the square root or arc sine was used.

For the open field test, just the data of latency movement was transformed with logarithmic and the vocalization was transformed with arc sine square root. Moreover, for the novel object test, the data of latency to reach the object was logarithmically transformed, and the data from exploratory behaviour and proximity of the object were square-root transformed.

The data of skin lesions and open field test behaviour were analysed using mixed models procedure of SAS (PROC MIXED) in a randomized block design.

The data of lesion scores were presented as untransformed least square means and SEM of pen averages. The data of open field and novel object test were presented as untransformed least square means and SEM on treatment averages.

Performance data were submitted to analysis of variance and the model included the effect of treatment as a fixed effect and the block as a random effect.

The means and SEM was obtained from raw data. For all parameters, a 5% level of significance was required.

## Results

Data on weight gain indicated that there was no treatment effect for the performance parameters evaluated (see S3 File). The performance data of the piglets are presented in Table 1. Litter weight at day 27^th^ of lactation, for LFD piglets, was numerically higher than HFD piglets, approaching significance.

**Table 1:** Performance measures obtained assessing data from 156 piglets born from 22 sows fed diets with 12.86% crude fibre (HFD; n = 14 sows; n = 100 piglets) or 2.53% crude fibre (LFD; n = 8 sows; n = 56 piglets), during lactation.

Variables	Treatment		SEM	P value
	HFD	LFD		
Number of piglets born alive	11.12	9.33	0.65	0.18
Number of piglets weaned	10.19	8.5	0.59	0.16
Litter weight 24 hours after birth (kg)	18.02	16.09	0.76	0.21
Average piglet weight 24 hours after birth (kg)	1.68	1.82	0.48	0.14
Average piglet weight at 21 days of lactation (kg)	6.34	6.72	0.21	0.39
Average piglet weight at 27 days of lactation (kg)	7.98	8.98	0.28	0.08
Average daily gain per litter (kg)	2.29	2.11	0.11	0.44
Average individual daily gain (kg)	0.24	0.25	0.01	0.43

Data collected by two independent evaluators revealed that piglets born from HFD sows had fewer skin lesions on the day of weaning than LFD piglets (HFD 4.78 lesions and LFD 7.14 lesions, P = 0.023; Table 2, see S1 File). No differences in skin lesions were observed on post-weaning days 1 (D29) and 2 (D30). Mixing of the animals, during weaning, induced an eight fold increase in the recorded skin lesions. The two independent observers analysed a total of 936 pictures and the difference between than was 1.6 lesions on D28, 3.8 lesions on D29 and 6.9 lesions on D30 (see S1 File). According to the Tukey-Kramer test, HFD and Mixed piglets were equal (P = 0.1236) and LFD piglets differed from HFD piglets (P = 0.0086) and Mixed piglets (P = 0.0016).

**Table 2:** Mean number of skin lesions per pen counted by two observers on photos of 156 piglets born from 22 sows fed diets with 12.86% crude fibre (HFD, n = 14 sows; n = 100 piglets) or 2.53% crude fibre (LFD; n = 8 sows; n = 56 piglets), during day 28 (prior to weaning) and days 29 and 30 (one and two days post-weaning, respectively).

Variable	Treatment			SEM	P value
	HFD	LFD	Mixed		
Number of skin lesions at D28	4.78	7.14	3.28	0.33	<0.01
Number of skin lesions at D29	31.01	42.66	30.99	2.44	0.27
Number of skin lesions at D30	25.70	29.87	27.36	1.59	0.70

The values shown are the average skin lesions per pen. HFD sows n = 21, LFD sows n = 10, Mixed n = 8. This results are the mean of the two evaluators

There was no effect of treatment on the behavioural measures obtained during the open field test and the novel object test (Tables 3 and 4). The number of vocalizations approached significance in the novel object test, being somewhat higher in the HFD piglets. The reason for this tendency could be a different way that piglets cope with challenges, as they were isolated from littermates and adopted different approaches in this stressful situation.

**Table 3:** Behaviour of piglets born from HFD (n = 87) or LFD (n = 55) sows in the open field test.

Variable	Treatment		Mean	SEM	P value
	HFD	LFD			
Latency movement (seconds)	6.78	4.32	5.95	0.68	0.12
Central quadrants (seconds)	29.91	30.50	29.31	0.91	0.77
Lateral quadrants (seconds)	60.65	59.85	57.49	2.02	0.85
Activity (seconds)	91.74	90.23	87.80	2.75	0.80
Number of vocalization (events)	202.92	171.89	190.81	8.54	0.34

**Table 4:** Behaviour of piglets born from HFD (n = 87) or LFD (n = 55) sows in the novel object test.

Variable	Treatment		Mean	SEM	P value
	HFD	LFD			
Latency to reach the object (seconds)	73.62	71.41	73.03	7.51	0.53
Exploratory behaviour (seconds)	16.14	14.83	15.33	0.99	0.93
Proximity to the object (seconds)	38.40	35.04	36.47	2.16	0.78
Number of vocalization (events)	195.10	163.85	184.91	7.83	0.07

## Discussion

The aim of the present study was to investigate the impact of a high fibre diet for pregnant gilts on the levels of skin lesions and behaviour in open field and novel object test. Piglets born from HFD sows had fewer skin lesions at the weaning day, day 28, than piglets form LFD sows. This may indicate that HFD piglets avoided engaging in mutual fights, or had less motivation to engage in aggressive interactions before weaning, and therefore had fewer skin lesions at weaning. Previous work demonstrated a correlation between agonistic behaviour and skin lesions in pigs [33]. Our data did not reveal any significant effect of dietary fibre content before weaning on skin lesions after weaning. At days 29 and 30 the skin lesions were considerably higher than at weaning in both groups, probably masking any treatment effect. It is important to highlight the unexpected finding that there was a difference in the mean skin lesions between the dietary fibre treatments at the weaning day, when, as we assumed, the piglets were still part of a stable social group. A likely cause of this difference could be differences between groups in competition to access milk. However, we did not identify any difference in the performance measures, such as total litter weight at birth, average weight at 21 days, average daily gain per litter and individual average individual daily gain. Only the average weight at 27 days was numerically higher for LFD piglets, when compared with HFD piglets, approaching significance (P = 0.077). The insignificantly higher average weights in LFD piglets at all ages may be related to slightly smaller litter sizes in the LFD group. Given the treatment effect on the weaning day, it was surprising to find no treatment effect in skin lesions on days 29 and 30, one and two days after weaning (see Table 2). The choice to house 4 animals per pen, two littermates, may have created an unnaturally less complex, social environment for recently weaned pigs. In natural settings, litters of 4–6 sows interact forming a rather complex social environment after weaning [36]. In commercial settings, the number of piglets housed in weaning pens varies, but it is seldom less the 12 animals. This methodology was adopted because of the importance to systematize the weaning of the piglets. It is plausible to argue that extremely challenging situations, as weaning more animals per pen as in commercial herds, could create a more challenging environment for animals, exacerbating the differences in agonistic behaviour observed before weaning.

The choice of open field and novel object tests, aimed at identifying characteristics of individual piglets, which could explain agonistic behaviour, measured by skin lesions. The behaviour of piglets in the open field test and novel object test showed no differences between treatments (Tables 3 and 4). The results found by [37] in rodents showed that a poor environment (without enrichment) before mating, generated more fearful offspring, which spent less time in the central quadrants in the open field test. In this study, the open field test and novel object test did not contribute to explaining the difference found in animal skin lesions before weaning period. Perhaps, with a large number of animals, we could test then in different ages to verify the correlation between the skin lesions prior to weaning and behaviour in open field and novel object tests. In addition, measuring the piglets behaviour, as well as the sow’s behaviour, during the suckling period could contribute to understanding deeply this difference in skin lesions.

## Conclusions

Our measures of lesions in piglets by weaning at 28 days of age, indicate that feeding pregnant sows with a HFD is effective in generating piglets that had fewer lesions prior to weaning, suggesting less agonistic interactions amongst littermates. This approach of HFD for sows in their piglets is novel and can be a viable alternative to alleviate aggressive behaviour among piglets during lactation. Data on the number of lesions and behavioural responses to the open field test and novel object test after weaning, did not identify differences between piglets of HFD or LFD sows. Further studies with long-term data collection on piglets and their mothers will provide valuable information about the consequences of diets in the prenatal period on the behaviour and welfare of piglets before and after weaning.

## Supporting Information

S1 File This is the S1 SKIN LESIONS.pdf. This file contain the information of all the skin lesion evaluated by two independent evaluators.
(PDF)

S2 File This is the S2 Open field and novel object test (1).pdf. This file contain all the information about emotionally tests.
(PDF)

S3 File This is the S3 PERFORMANCE.pdf. This file contain all data of performance measures.
(PDF)

## References

[1] Petherick JC, Blackshaw JK. A review of the factors influencing the aggressive and agonistic behaviour of the domestic pig. Aust J Exp Agric. 1987;27: 605–611.

[2] Camerlink I., Turner S. P., Ursinus W. W., Reimert I., & Bolhuis J. E. (2014). Aggression and affiliation during social conflict in pigs. PLoS ONE, 9(11), 1–21. 10.1371/journal.pone.0113502 PMC4245130 25427249

[3] Poltyrev T, Keshet GI, Kay G, Weinstock M. Role of experimental conditions in determining differences in exploratory behavior of prenatally stressed rats. Dev Psychobiol. John Wiley & Sons, Inc.; 1996;29: 453–462. 2-N 10.1002/(SICI)1098-2302(199607)29:5&lt;453::AID-DEV4&gt;3.0.CO;2-N 8809495

[4] Brunton PJ. Effects of maternal exposure to social stress during pregnancy: Consequences for mother and offspring. Reproduction. 2013;146. 10.1530/REP-13-0258 23901130

[5] Janczak AM, Pedersen LJ, Bakken M. Aggression, fearfulness and coping styles in female pigs. Appl Anim Behav Sci. 2003;81: 13–28.

[6] Tuchscherer M, Kanitz E, Otten W, Tuchscherer A. Effects of prenatal stress on cellular and humoral immune responses in neonatal pigs. Vet Immunol Immunopathol. 2002;86: 195–203. 12007885 10.1016/s0165-2427(02)00035-1

[7] Couret D, Prunier A, Mounier A-M, Thomas F, Oswald IP, Merlot E. Comparative effects of a prenatal stress occurring during early or late gestation on pig immune response. Physiol Behav. Elsevier Inc.; 2009;98: 498–504. 10.1016/j.physbeh.2009.08.003 19686768

[8] Rutherford KMD, Piastowska-Ciesielska A, Donald RD, Robson SK, Ison SH, Jarvis S, et al Prenatal stress produces anxiety prone female offspring and impaired maternal behaviour in the domestic pig. Physiol Behav. Elsevier Inc.; 2014;129: 255–264. 10.1016/j.physbeh.2014.02.052 24631303

[9] Yuan Y, Jansen J, Charles D, Zanella AJ. The influence of weaning age on post-mixing agonistic interactions in growing pigs. Appl Anim Behav Sci. 2004;88: 39–46.

[10] Souza AS, Jansen J, Tempelman RJ, Mendl M, Zanella AJ. A novel method for testing social recognition in young pigs and the modulating effects of relocation. Appl Anim Behav Sci. 2006;99: 77–87.

[11] Jarvis S, Moinard C, Robson SK, Baxter E, Ormandy E, Douglas AJ, et al Programming the offspring of the pig by prenatal social stress: Neuroendocrine activity and behaviour. Horm Behav. 2006;49: 68–80. 10.1016/j.yhbeh.2005.05.004 15961089

[12] Coulon M, Wellman CL, Marjara IS, Janczak AM, Zanella AJ. Early adverse experience alters dendritic spine density and gene expression in prefrontal cortex and hippocampus in lambs. Psychoneuroendocrinology. Elsevier; 2013;38: 1112–21. 10.1016/j.psyneuen.2012.10.018 23265310

[13] Petit B, Boissy A, Zanella A, Chaillou E, Andanson S, Bes S, et al Stress during pregnancy alters dendritic spine density and gene expression in the brain of new-born lambs. Behav Brain Res. 2015;291: 155–63. 10.1016/j.bbr.2015.05.025 26005125

[14] Andersen IL, Andenæs H, Bøe KE, Jensen P, Bakken M. The effects of weight asymmetry and resource distribution on aggression in groups of unacquainted pigs. Appl Anim Behav Sci. 2000;68: 107–120. 10771319 10.1016/s0168-1591(00)00092-7

[15] Morgan C., Deans L., Lawrence A., Nielsen B. The effects of straw bedding on the feeding and social behaviour of growing pigs fed by means of single-space feeders. Appl Anim Behav Sci. 1998;58: 23–33.

[16] Turner SP, Ewen M, Rooke JA, Edwards SA. The effect of space allowance on performance, aggression and immune competence of growing pigs housed on straw deep-litter at different group sizes. Livest Prod Sci. 2000;66: 47–55.

[17] Algers B, Blokhuis HJ, Broom DM, Costa P, Domingo M, Guemene D, et al Animal health and welfare aspects of different housing and husbandry systems for adult breeding boars, pregnant, farrowing Scientific Opinion of the Panel on Animal Health and Welfare Adopted on 10 October 2007. EFSA J. 2007;572: 1–13.

[18] De Leeuw JA, Zonderland JJ, Altena H, Spoolder HAM, Jongbloed AW, Verstegen MWA. Effects of levels and sources of dietary fermentable non-starch polysaccharides on blood glucose stability and behaviour of group-housed pregnant gilts. Appl Anim Behav Sci. 2005;94: 15–29.

[19] De Leeuw JA, Jongbloed AW, Verstegen MWA. Nutrient Metabolism Dietary Fiber Stabilizes Blood Glucose and Insulin Levels and Reduces Physical Activity in Sows (Sus scrofa). J Nutr. 2004;134: 1481–1486 15173415 10.1093/jn/134.6.1481

[20] Zonderland JJ, De Leeuw JA, Nolten C, Spoolder HAM. Assessing long-term behavioural effects of feeding motivation in group-housed pregnant sows; What, when and how to observe. Appl Anim Behav Sci. 2004;87: 15–30.

[21] Lawrence AB, Appleby MC, Macleod HA. Measuring hunger in the pig using operant conditioning: The effect of food restriction. Anim Prod. 1988;47: 131–137.

[22] Appleby MC, Lawrence AB. Food Restriction as a Cause of Stereotypic Behaviour in Tethered Gilts. Anim Prod. 1987;45: 103–110.

[23] Lawrence A. B.; Terlouw E. M. A review of behavioral factors involved in the development and continued performance of stereotypic behavior in pigs. J Anim Sci. 1993:71: 2815–2825 8226385 10.2527/1993.71102815x

[24] Meunier-Salaün MC, Edwards SA, Robert S. Effect of dietary fibre on the behaviour and health of the restricted fed sow. Anim Feed Sci Technol. 2001;90: 53–69.

[25] Gentilini FP, Dallanora D, Peixoto CH, Bernardi ML, Wentz I, Bortolozzo FP. Desempenho produtivo de leitoas alimentadas com dietas de gestação de baixo ou alto nível de casca de soja. Ciência Rural. 2004;34: 1177–1183

[26] Bolhuis JE, van den Brand H, Bartels AC, Oostindjer M, van den Borne JJGC, Kemp B, et al Effects of fermentable starch on behaviour of growing pigs in barren or enriched housing. Appl Anim Behav Sci. 2010;123: 77–86.

[27] Robert S, Matte JJ, Farmer C, Girard CL, Martineau GP. High-fibre diets for sows: effects on stereotypies and adjunctive drinking. Appl Anim Behav Sci. 1993;37: 297–309.

[28] Brouns F, Edwards SA, English PR. Effect of dietary fiber and feeding system on activity and oral behavior of group-housed gilts. Appl Anim Behav Sci. 1994;39: 215–223.

[29] Lepionka L, Malbert CH, Laplace J. Proximal gastric distension modifies ingestion rate in pigs. Reprod Nutr Dev. 1997;37: 449–457. 9342794 10.1051/rnd:19970406

[30] Rérat A. Influence of the nature of carbohydrate intake on the absorption chronology of reducing sugars and volatile fatty acids in the pig. Reprod Nutr Dev. 1996;36: 3–19. 8881588 10.1051/rnd:19960101

[31] Holt JP, Johnston LJ, Baidoo SK, Shurson GC. Effects of a high-fiber diet and frequent feeding on behavior, reproductive performance, and nutrient digestibility in gestating sows. J Anim Sci. 2006;84: 946–955 16543573 10.2527/2006.844946x

[32] Veum TL, Crenshaw JD, Crenshaw TD, Cromwell GL, Easter RA, Ewan RC, et al The addition of ground wheat straw as a fiber source in the gestation diet of sows and the effect on sow and litter performance for three successive parities. J Anim Sci. 2009;87: 1003–1012. 10.2527/jas.2008-1119 18952734

[33] Turner SP, Farnworth MJ, White IMS, Brotherstone S, Mendl M, Knap P, et al The accumulation of skin lesions and their use as a predictor of individual aggressiveness in pigs. Appl Anim Behav Sci. 2006;96: 245–259.

[34] Guy JH, Burns SE, Barker JM, Edwards SA. Reducing post-mixing aggression and skin lesions in weaned pigs by application of a synthetic maternal pheromone. Anim Welf. 2009;18: 249–255

[35] Puppe B, Ernst K, Schön PC, Manteuffel G. Cognitive enrichment affects behavioural reactivity in domestic pigs. Appl Anim Behav Sci. 2007;105: 75–86.

[36] Harris MJ, Bergeron R, Gonyou HW. Parturient behaviour and offspring-directed aggression in farmed wild boar of three genetic lines. Appl Anim Behav Sci. 2001;74: 153–163.

[37] Dell PA, Rose FD. Transfer of effects from environmentally enriched and impoverished female rats to future offspring. Physiol Behav. 1987;39: 187–190. 3575452 10.1016/0031-9384(87)90008-4

