# Supplementary material for: Piglets Born from Sows Fed High Fibre Diets during Pregnancy Are Less Aggressive Prior to Weaning
Source: PLoS One. 2016 Dec 1;11(12):e0167363. doi: 10.1371/journal.pone.0167363 (PMC5132218; doi:10.1371/journal.pone.0167363)
Supplement: S1 File — This file contain the information of all the skin lesion evaluated by two independent evaluators. (PDF) [file pone.0167363.s001.pdf]

	D28		
IDENTIFICATION	OBSEVER 1	OBSERVER 2	MEDIA 1 and 2
146	1	0	0,5
147	5	4	4,5
149	9	10	9,5
150	0	0	0
152	0	0	0
153	3	2	2,5
154	2	3	2,5
155	0	6	3
133	8	4	6
134	0	1	0,5
135	7	3	5
136	3	3	3
138	1	0	0,5
139	2	0	1
140	2	1	1,5
141	4	4	4
160	7	3	5
161	14	13	13,5
164	11	10	10,5
165	10	9	9,5
167	11	4	7,5
168	6	6	6
171	13	9	11
172	17	12	14,5
174	11	9	10
176	3	7	5
177	1	3	2
178	1	6	3,5
181	0	0	0
183	6	2	4
184	1	4	2,5
186	6	1	3,5
187	2	1	1,5
188	1	2	1,5
189	0	0	0
190	0	0	0
192	0	0	0
193	8	6	7
194	3	2	2,5
198	0	0	0
199	5	7	6
200	2	2	2
202	1	0	0,5
204	1	0	0,5
205	6	4	5

206	3	1	2
207	13	8	10,5
208	5	3	4
285	3	0	1,5
286	1	0	0,5
287	6	5	5,5
292	4	6	5
294	0	1	0,5
295	3	4	3,5
296	1	6	3,5
297	5	1	3
273	8	1	4,5
274	6	2	4
275	0	0	0
276	0	0	0
278	1	3	2
279	1	2	1,5
280	5	2	3,5
281	7	2	4,5
299	13	8	10,5
300	6	6	6
301	27	20	23,5
303	3	0	1,5
304	6	3	4,5
306	10	3	6,5
331	.	2	2
332	.	2	2
333	.	0	0
334	.	1	1
336	.	0	0
337	.	1	1
338	20	17	18,5
339	0	1	0,5
340	6	1	3,5
341	11	9	10
342	2	3	2,5
344	17	8	12,5
318	6	3	4,5
319	1	1	1
320	1	1	1
323	0	1	0,5
324	12	1	6,5
326	0	1	0,5
355	16	6	11
359	17	8	12,5
361	8	3	5,5
362	4	0	2

363	4	3	3,5
364	3	1	2
398	2	2	2
399	7	4	5,5
400	2	3	2,5
402	3	1	2
403	10	3	6,5
404	0	0	0
348	2	2	2
349	5	5	5
351	6	4	5
352	2	2	2
353	14	11	12,5
354	5	4	4,5
377	24	8	16
381	4	2	3
382	1	0	0,5
386	11	7	9
387	6	6	6
388	3	2	2,5
408	5	3	4
409	2	1	1,5
410	7	8	7,5
411	5	3	4
412	5	5	5
413	1	1	1
414	3	2	2,5
415	1	0	0,5
427	3	3	3
428	2	3	2,5
429	2	3	2,5
431	8	11	9,5
433	0	5	2,5
434	3	3	3
435	3	5	4
436	3	4	3,5
499	3	0	1,5
500	1	3	2
504	0	5	2,5
506	1	2	1,5
508	4	2	3
509	0	1	0,5
512	0	1	0,5
515	9	6	7,5
516	7	5	6
517	14	15	14,5
519	28	25	26,5

520	10	6	8
525	10	8	9
526	5	6	5,5
528	8	7	7,5
530	4	1	2,5
533	11	5	8
534	24	10	17
535	10	6	8
536	7	9	8
537	3	2	2,5
538	5	3	4
539	0	0	0
540	11	5	8
543	1	0	0,5
544	1	2	1,5
545	4	2	3
546	3	2	2,5

	D29		
IDENTIFICATION	OBSEVER 1	OBSERVER 2	MEDIA 1 and 2
146	19	17	18
147	27	23	25
149	30	29	29,5
150	40	28	34
152	2	2	2
153	50	38	44
154	36	35	35,5
155	84	55	69,5
133	70	53	61,5
134	45	34	39,5
135	69	44	56,5
136	10	32	21
138	59	56	57,5
139	1	4	2,5
140	41	36	38,5
141	0	3	1,5
160	37	40	38,5
161	81	44	62,5
164	40	28	34
165	20	24	22
167	76	53	64,5
168	44	33	38,5
171	22	16	19
172	42	36	39
174	26	25	25,5
176	42	42	42

177	12	15	13,5
178	22	20	21
181	2	8	5
183	25	21	23
184	23	27	25
186	22	27	24,5
187	46	41	43,5
188	2	4	3
189	12	15	13,5
190	10	9	9,5
192	9	8	8,5
193	42	25	33,5
194	3	2	2,5
198	13	8	10,5
199	108	98	103
200	27	32	29,5
202	25	30	27,5
204	6	8	7
205	41	42	41,5
206	21	25	23
207	18	22	20
208	1	1	1
285	4	6	5
286	20	16	18
287	13	13	13
292	0	2	1
294	11	10	10,5
295	42	36	39
296	31	23	27
297	3	1	2
273	36	31	33,5
274	28	29	28,5
275	18	14	16
276	35	28	31,5
278	44	26	35
279	23	21	22
280	112	71	91,5
281	1	2	1,5
299	72	63	67,5
300	66	51	58,5
301	33	25	29
303	97	69	83
304	55	50	52,5
306	20	20	20
331	0	21	10,5
332	0	26	13
333	0	7	3,5

334	0	34	17
336	0	51	25,5
337	0	46	23
338	48	44	46
339	40	34	37
340	38	35	36,5
341	46	42	44
342	60	68	64
344	40	42	41
318	69	47	58
319	65	48	56,5
320	41	27	34
323	33	23	28
324	79	41	60
326	44	34	39
355	43	33	38
359	39	27	33
361	7	7	7
362	3	2	2,5
363	7	9	8
364	6	6	6
398	30	23	26,5
399	12	9	10,5
400	10	9	9,5
402	14	12	13
403	9	7	8
404	6	10	8
348	7	16	11,5
349	60	56	58
351	2	4	3
352	25	29	27
353	79	55	67
354	30	24	27
377	62	52	57
381	4	6	5
382	45	35	40
386	31	30	30,5
387	51	48	49,5
388	19	23	21
408	94	65	79,5
409	33	22	27,5
410	69	46	57,5
411	72	37	54,5
412	80	52	66
413	24	26	25
414	10	15	12,5
415	41	30	35,5

427	63	53	58
428	12	17	14,5
429	13	11	12
431	61	46	53,5
433	41	36	38,5
434	76	53	64,5
435	23	18	20,5
436	70	57	63,5
499	17	17	17
500	24	26	25
504	27	27	27
505	41	44	42,5
506	27	32	29,5
508	26	23	24,5
509	24	22	23
512	.	22	22
515	.	.	
516	17	21	19
517	46	53	49,5
519	80	63	71,5
520	34	36	35
525	53	55	54
526	9	8	8,5
528	17	28	22,5
530	38	41	39,5
533	70	62	66
534	39	49	44
535	34	42	38
536	39	41	40
537	70	43	56,5
538	34	29	31,5
539	15	10	12,5
540	23	18	20,5
543	10	11	10,5
544	23	17	20
545	30	22	26
546	21	14	17,5

D30

IDENTIFICATION	OBSEVER 1	OBSERVER 2	MEDIA 1 and 2
146	33	23	28
147	23	21	22
149	31	34	32,5
150	33	26	29,5
152	2	5	3,5

153	54	34	44
154	16	11	13,5
155	38	28	33
133	49	47	48
134	16	19	17,5
135	41	31	36
136	16	15	15,5
138	57	40	48,5
139	2	5	3,5
140	29	33	31
141	8	6	7
160	43	44	43,5
161	38	41	39,5
164	31	26	28,5
165	17	20	18,5
167	41	34	37,5
168	25	26	25,5
171	19	20	19,5
172	45	33	39
174	23	20	21,5
176	40	26	33
177	16	10	13
178	15	11	13
181	2	5	3,5
183	26	24	25
184	20	19	19,5
186	24	26	25
187	55	41	48
188	12	11	11,5
189	11	10	10,5
190	17	13	15
192	6	7	6,5
193	34	19	26,5
194	23	21	22
198	22	18	20
199	98	72	85
200	41	35	38
202	18	18	18
204	20	16	18
205	57	37	47
206	19	16	17,5
207	19	17	18
208	11	15	13
285	4	1	2,5
286	12	8	10
287	19	19	19
292	0	1	0,5

294	10	7	8,5
295	21	20	20,5
296	25	26	25,5
297	25	23	24
273	23	14	18,5
274	10	13	11,5
275	19	11	15
276	31	25	28
278	36	16	26
279	22	20	21
280	59	35	47
281	21	13	17
299	80	51	65,5
300	45	42	43,5
301	56	47	51,5
303	61	52	56,5
304	62	44	53
306	33	18	25,5
331	.	18	18
332	.	17	17
333	.	6	6
334	.	29	29
336	.	34	34
337	.	23	23
338	36	24	30
339	41	31	36
340	40	24	32
341	26	23	24,5
342	37	26	31,5
344	18	15	16,5
318	39	33	36
319	53	49	51
320	18	18	18
323	24	12	18
324	34	20	27
326	20	24	22
355	21	13	17
359	29	22	25,5
361	7	7	7
362	8	10	9
363	15	8	11,5
364	0	3	1,5
398	26	19	22,5
399	19	9	14
400	24	6	15
402	14	6	10
403	14	7	10,5

404	14	12	13
348	2	1	1,5
349	41	26	33,5
351	8	5	6,5
352	26	17	21,5
353	69	41	55
354	33	23	28
377	54	43	48,5
381	5	6	5,5
382	32	26	29
386	21	24	22,5
387	34	28	31
388	16	15	15,5
408	36	18	27
409	18	16	17
410	54	27	40,5
411	55	33	44
412	53	29	41
413	37	27	32
414	11	9	10
415	33	22	27,5
427	41	35	38
428	13	10	11,5
429	20	14	17
431	27	24	25,5
433	30	30	30
434	50	39	44,5
435	17	18	17,5
436	54	36	45
499	20	18	19
500	18	14	16
504	49	31	40
506	21	24	22,5
508	31	24	27,5
509	31	26	28,5
512	9	15	12
515	36	22	29
516	21	18	19,5
517	48	33	40,5
519	79	53	66
520	41	41	41
525	59	42	50,5
526	5	10	7,5
528	28	20	24
530	41	25	33
533	57	43	50
534	32	24	28

535	41	37	39
536	42	36	39
537	96	61	78,5
538	41	34	37,5
539	35	20	27,5
540	28	22	25
543	18	15	16,5
544	31	20	25,5
545	29	19	24
546	35	25	30
