# Supplementary material for: Piglets Born from Sows Fed High Fibre Diets during Pregnancy Are Less Aggressive Prior to Weaning
Source: PLoS One. 2016 Dec 1;11(12):e0167363. doi: 10.1371/journal.pone.0167363 (PMC5132218; doi:10.1371/journal.pone.0167363)
Supplement: S2 File — This file contain all the information about emotionally tests. (PDF) [file pone.0167363.s002.pdf]

				OPEN FIELD			
**IDENTIFICATION**	TREATMENT	**LATENCY**	**CENTRAL QUADRANTS**	**LATERAL QUADRANTS**	**ACTIVITY**	**VOCALIZATIONS**	**BLOCK**
**134**	HFD	18,64	14	21	35	21	1
**135**	HFD	1,66	27	50	77	83	1
**136**	HFD	18.37	10	29	39	41	1
**138**	HFD	25,41	25	80	113	144	1
**140**	HFD	6,75	19	54	73	87	1
**146**	HFD	22,37	18	31	49	292	1
**147**	HFD	4,28	23	66	89	350	1
**149**	HFD	3,00	26	68	94	309	1
**150**	HFD	51,75	12	12	24	38	1
**152**	HFD	7,13	25	57	82	180	1
**153**	HFD	36,29	33	31	64	193	1
**154**	HFD	28,35	30	54	84	88	1
**155**	HFD	3,60	24	72	96	407	1
**160**	LFD	0,00	63	118	181	158	1
**161**	LFD	8,16	38	92	130	185	1
**164**	LFD	8,50	22	47	69	36	1
**165**	LFD	1,68	40	69	109	194	1
**167**	LFD	0,00	25	68	93	213	1
**168**	LFD	3,46	32	73	105	260	1
**171**	LFD	2,06	40	72	112	180	1
**172**	LFD	5,63	38	102	140	229	1
**174**	LFD	2,22	43	90	133	257	1
**176**	LFD	7,28	21	45	66	213	1
**177**	LFD	21,34	29	78	107	139	1
**178**	LFD	17,78	36	57	93	96	1
**181**	LFD	37,28	15	26	41	84	1
**183**	LFD	3,85	38	150	118	163	1
**184**	LFD	5,22	20	28	48	4	1
**186**	LFD	3,37	17	28	45	85	1
**187**	HFD	0,00	48	57	105	228	1
**188**	HFD	1,22	17	30	47	137	1

**189**	HFD	1,03	34	29	63	368	1
**190**	HFD	0,00	28	42	70	327	1
**192**	HFD	1,28	36	32	68	169	1
**193**	HFD	2,81	40	89	129	288	1
**194**	HFD	2,28	25	57	82	236	1
**198**	HFD	3,00	38	71	109	280	1
**199**	LFD	2,91	46	48	94	201	1
**200**	LFD	5,78	22	79	101	275	1
**202**	LFD	5,90	34	45	79	245	1
**205**	LFD	3,47	25	32	57	389	1
**206**	LFD	1,34	34	86	120	271	1
**207**	LFD	7,10	22	84	106	210	1
**208**	LFD	0,00	39	46	85	213	1
**274**	HFD	0,00	34	63	97	107	2
**276**	HFD	2,34	33	72	105	209	2
**278**	HFD	6,60	36	84	120	98	2
**279**	HFD	1,65	27	63	90	278	2
**280**	HFD	0,00	42	89	131	347	2
**281**	HFD	2,81	38	92	130	325	2
**285**	HFD	1,09	12	44	56	57	2
**286**	HFD	11,63	27	58	85	225	2
**287**	HFD	4,97	30	88	118	347	2
**292**	HFD	3,25	27	48	75	121	2
**294**	HFD	8,84	29	49	78	194	2
**295**	HFD	6,25	31	85	116	330	2
**296**	HFD	11,03	20	34	54	198	2
**297**	HFD	3,93	26	69	95	294	2
**299**	LFD	3,03	19	40	59	57	2
**300**	LFD	8,88	32	54	86	122	2
**301**	LFD	1,03	29	58	87	241	2
**303**	LFD	2,59	30	73	103	268	2
**304**	LFD	8,75	15	51	66	65	2
**306**	LFD	7,94	25	43	68	135	2

**318**	HFD	0,00	32	45	77	139	2
**319**	HFD	5,56	23	36	59	130	2
**320**	HFD	1,75	28	68	96	270	2
**323**	HFD	12,22	37	79	116	293	2
**324**	HFD	7,31	34	85	119	255	2
**326**	HFD	0,00	20	60	80	251	2
**331**	LFD	3,63	19	38	57	182	2
**332**	LFD	4,81	35	85	120	287	2
**333**	LFD	3,21	21	35	56	89	2
**334**	LFD	0,00	44	63	107	193	2
**336**	LFD	6,00	30	42	72	292	2
**337**	LFD	3,34	24	51	75	327	2
**338**	HFD	6,41	31	66	97	152	2
**339**	HFD	8,69	40	44	84	340	2
**340**	HFD	16,25	38	138	176	332	2
**341**	HFD	0,00	56	93	149	456	2
**342**	HFD	0,00	30	26	56	283	2
**344**	HFD	1,40	45	50	105	105	2
**348**	LFD	0,00	43	39	82	46	2
**349**	LFD	0,00	22	38	60	54	2
**351**	LFD	26,12	3	11	14	17	2
**352**	LFD	0,00	37	72	109	42	2
**353**	LFD	3,53	26	41	67	140	2
**354**	LFD	9,28	20	30	50	53	2
**355**	HFD	1,29	24	43	67	136	2
**359**	HFD	4,79	13	38	51	124	2
**361**	HFD	1,84	31	59	90	72	2
**362**	HFD	3,25	20	47	67	198	2
**363**	HFD	6,66	8	31	39	38	2
**364**	HFD	4,69	29	44	73	47	2
**377**	LFD	5,47	13	63	76	55	2
**381**	LFD	6,81	29	42	71	147	2
**382**	LFD	0,00	15	21	36	62	2

**386**	LFD	2,03	26	41	67	225	2
**387**	LFD	1,38	34	58	92	190	2
**388**	LFD	0,00	45	71	116	220	2
**398**	HFD	0,00	39	83	122	293	2
**399**	HFD	0,00	16	26	42	22	2
**400**	HFD	0,00	18	45	63	254	2
**402**	HFD	14,44	33	74	107	180	2
**403**	HFD	2,00	26	44	70	183	2
**404**	HFD	8,46	30	48	78	292	2
**408**	LFD	0,00	27	43	70	299	2
**409**	LFD	0,00	30	38	68	230	2
**410**	LFD	0,00	33	47	80	236	2
**411**	LFD	4,03	32	57	89	296	2
**412**	LFD	3,62	25	57	82	77	2
**413**	LFD	0,00	32	76	108	94	2
**414**	LFD	0,00	22	29	51	239	2
**415**	LFD	5,90	30	60	90	174	2
**427**	HFD	1,16	41	69	110	251	2
**428**	HFD	0,00	24	78	102	164	2
**429**	HFD	8,91	10	28	38	88	2
**431**	HFD	23,22	32	35	67	66	2
**433**	HFD	4,81	21	82	103	189	2
**434**	HFD	14,25	26	33	59	122	2
**435**	HFD	13,68	19	14	53	28	2
**436**	HFD	0,00	19	41	60	246	2
**499**	HFD	3,75	46	113	159	360	3
**500**	HFD	1,34	52	92	144	298	3
**504**	HFD	0,00	25	54	79	206	3
**506**	HFD	0,00	44	87	131	198	3
**508**	HFD	1,32	30	79	109	100	3
**509**	HFD	0,00	26	90	116	258	3
**512**	HFD	4,25	12	24	36	76	3
**515**	HFD	0,00	55	106	161	373	3

**516**	HFD	1,81	36	75	111	132	3
**517**	HFD	1,46	49	118	167	249	3
**519**	HFD	4,46	15	45	60	53	3
**520**	HFD	22,44	12	43	55	96	3
**528**	HFD	9,31	36	77	113	240	3
**530**	HFD	7,31	16	25	41	76	3
**534**	HFD	2,82	55	128	183	177	3
**536**	HFD	11,40	24	55	79	379	3
**537**	HFD	3,09	35	39	74	181	3
**539**	HFD	1,00	45	53	98	378	3
**544**	HFD	6,19	27	46	73	84	3
**545**	HFD	4,40	46	104	150	164	3

		NOVEL OBJECT			
	**LATENCY**	**EXPLORATORY BEHAVIOUR**	**PROXIMITY TO OBJECT**	**VOCALIZATION**	**BLOCK**
HFD	0,00	68	94	147	1
HFD	3,30	26	40	107	1
HFD	140,00	25	36	162	1
HFD	3,60	29	57	158	1
HFD	13,90	14	18	194	1
HFD	0,00	21	50	277	1
HFD	1,06	8	26	262	1
HFD	5,60	16	37	341	1
HFD	48,20	6	15	204	1
HFD	5,40	71	108	253	1
HFD	67,50	30	53	400	1
HFD	1,00	28	46	178	1
HFD	45,30	17	37	299	1
LFD	5,90	29	78	192	1
LFD	8,19	12	32	103	1
LFD	31,06	20	39	119	1
LFD	4,15	21	48	197	1
LFD	4,19	29	45	196	1
LFD	6,94	28	57	297	1
LFD	126,13	8	13	196	1
LFD	3,41	18	30	259	1
LFD	5,47	11	54	238	1
LFD	240,09	7	9	67	1
LFD	47,13	18	50	126	1
LFD	23,38	21	40	92	1
LFD	1,63	19	45	170	1
LFD	130,25	33	59	84	1
LFD	92,47	12	18	71	1
LFD	9,25	17	50	98	1
HFD	2,04	25	52	169	1
HFD	209,85	1	15	96	1

HFD	81,15	21	115	357	1
HFD	3,69	3	16	264	1
HFD	45,06	11	61	383	1
HFD	0,00	23	57	293	1
HFD	30,32	26	91	101	1
HFD	25,22	21	51	353	1
LFD	13,82	16	67	244	1
LFD	0,00	28	61	258	1
LFD	128,66	21	59	212	1
LFD	4,53	18	40	379	1
LFD	15,00	27	64	177	1
LFD	13,40	28	45	196	1
LFD	28,34	11	49	198	1
HFD	132,49	8	29	191	2
HFD	5,53	9	38	116	2
HFD	75,19	10	28	99	2
HFD	35,91	40	70	190	2
HFD	17,75	9	60	229	2
HFD	9,88	20	71	165	2
HFD	50,35	9	19	199	2
HFD	34,41	11	19	221	2
HFD	9,22	20	87	242	2
HFD	300,00	0	0	28	2
HFD	25,97	11	17	301	2
HFD	50,06	15	37	257	2
HFD	8,63	18	24	191	2
HFD	146,13	3	7	234	2
LFD	122,53	8	14	22	2
LFD	11,25	16	34	195	2
LFD	2,72	23	44	240	2
LFD	29,53	15	40	183	2
LFD	14,12	11	19	47	2
LFD	89,29	23	31	53	2

HFD	30,56	13	35	139	2
HFD	25,06	15	35	124	2
HFD	33,78	7	26	268	2
HFD	87,09	8	16	159	2
HFD	27,50	20	108	162	2
HFD	24,32	7	22	350	2
LFD	213,67	10	16	173	2
LFD	14,22	14	29	234	2
LFD	48,34	15	38	176	2
LFD	43,13	13	28	241	2
LFD	35,94	22	41	263	2
LFD	0,00	12	32	295	2
HFD	35,34	4	8	130	2
HFD	38,81	7	14	336	2
HFD	24,66	20	56	282	2
HFD	32,82	5	8	349	2
HFD	67,09	26	73	158	2
HFD	14,56	12	21	147	2
LFD	74,28	5	11	90	2
LFD	93,84	7	18	89	2
LFD	86,75	8	31	77	2
LFD	16,43	18	23	70	2
LFD					2
LFD	76,88	5	7	142	2
HFD	71,91	11	17	155	2
HFD	16,16	33	57	78	2
HFD	20,22	15	39	75	2
HFD	186,25	0	15	40	2
HFD	67,28	12	16	53	2
HFD	300,00	0	0	8	2
LFD	264,04	1	3	60	2
LFD	228,87	0	8	161	2
LFD	300,00	0	0	48	2

LFD	119,66	10	24	238	2
LFD	72,07	19	40	168	2
LFD	30,81	25	46	75	2
HFD	21,28	37	85	237	2
HFD	300,00	0	0	20	2
HFD	494,40	6	6	351	2
HFD	11,50	11	30	201	2
HFD	24,16	22	63	197	2
HFD	300,00	0	0	135	2
LFD	74,24	13	35	287	2
LFD	31,69	13	33	197	2
LFD	160,35	7	14	156	2
LFD	41,40	2	5	384	2
LFD	97,28	91	126	56	2
LFD	8,84	26	37	177	2
LFD	133,09	0	10	256	2
LFD	116,97	23	55	185	2
HFD	6,09	3	11	239	2
HFD	29,03	5	14	230	2
HFD	28,25	15	19	100	2
HFD	195,03	9	11	315	2
HFD	72,96	40	58	194	2
HFD	274,37	5	5	340	2
HFD	176,72	66	75	47	2
HFD	91,06	7	20	277	2
HFD	15,18	3	17	331	3
HFD	125,56	28	45	146	3
HFD	138,62	13	30	195	3
HFD	6,56	21	98	175	3
HFD	51,90	10	32	92	3
HFD	97,43	6	17	213	3
HFD	102,59	12	46	146	3
HFD	27,53	39	49	312	3

HFD	12,25	9	21	49	3
HFD	20,66	7	17	98	3
HFD	300,00	0	0	78	3
HFD	35,48	27	40	171	3
HFD	24,96	8	33	213	3
HFD	248,34	4	5	146	3
HFD	15,75	21	51	121	3
HFD	21,65	35	52	341	3
HFD	184,41	6	28	63	3
HFD	53,00	10	59	269	3
HFD	157,22	8	14	41	3
HFD	300,00	0	0	109	3
