# Supplementary material for: Piglets Born from Sows Fed High Fibre Diets during Pregnancy Are Less Aggressive Prior to Weaning
Source: PLoS One. 2016 Dec 1;11(12):e0167363. doi: 10.1371/journal.pone.0167363 (PMC5132218; doi:10.1371/journal.pone.0167363)
Supplement: S3 File — This file contain all data of performance measures. (PDF) [file pone.0167363.s003.pdf]

IDENTIFICATION	TREATMENT	GESTATION LENGH	BORN ALIVE	WEANED	CRUSHED	LITTER WEIGHT 24 HOURS AFTER BIRTH	AVERAGE PIGLET WEIGHT 24 HOURS AFTER BIRTH	AVERAGE PIGLET WEIGHT AT 21 DAYS OF LACTATION
4	HFD	115	14	13	1	18,4	1,415384615	5,484615385
5	HFD	114	9	7	2	14,65	2,092857143	6,342857143
6	HFD	115	12	11	0	19,58	1,631666667	6,566666667
9	HFD	114	11	9	2	17,53	1,593636364	7,477777778
10	HFD	114	12	12	0	20,45	1,704166667	6,233333333
11	HFD	113	3	3	0	5,8	1,933333333	9,433333333
17	HFD	117	15	13	2	23,1	1,776923077	5,892307692
20	HFD	113	14	13	1	20,5	1,576923077	5,084615385
21	HFD	113	13	11	2	18,45	1,419230769	6,681818182
22	HFD	113	12	12	0	19,52	1,626666667	5,125
24	HFD	116	14	12	2	19,29	1,6075	5,35
25	HFD	114	7	7	0	13,05	1,864285714	7,642857143
28	HFD	115	12	11	1	17,3	1,572727273	5,872727273
33	HFD	114	13	12	1	19,75	1,645833333	5,466666667
35	HFD	115	10	10	0	16,27	1,627	5,58
36	HFD	115	7	7	0	12,4	1,771428571	7,242857143
3	LFD	111	13	12	1	18	1,384615385	5,853846154
7	LFD	113	13	12	0	19,2	1,476923077	6,453846154
8	LFD	114	9	9	0	16,18	1,797777778	8,2
13	LFD	113	5	5	6	8,95	1,79	7,36
14	LFD	116	12	12	0	21,1	1,758333333	6,266666667
15	LFD	117	9	7	1	15,3	1,9125	5,975
23	LFD	114	9	8	1	14,95	1,86875	7,1375
26	LFD	113	10	10	2	18,7	1,558333333	5,08
27	LFD	111	3	3	0	7,15	2,383333333	8,2
29	LFD	115	5	5	0	11,05	2,21	8
30	LFD	115	9	6	3	20,25	2,25	6,683333333
32	LFD	113	15	13	0	22,27	1,484666667	5,413333333

AVERAGE PIGLET WEIGHT AT 27 DAYS OF LACTATION	AVERAGE DAILY GAIN PER LITTER	AVERAGE INDIVIDUAL DAILY GAIN
6,769230769	2,4	0,1846
7,455555556	1,8732	0,2676
7,616666667	2,565	0,2138
9,377777778	2,4767	0,2752
8,341666667	2,7465	0,2289
.	1,071	0,3571
7,961538462	2,871	0,2209
6,530769231	2,9196	0,2654
9,109090909	2,9196	0,2654
7,125	2,0619	0,1718
6,666666667	1,9584	0,1632
10,01428571	2,0375	0,2911
8,136363636	2,329	0,2117
6,875	2,2411	0,1868
7,89	2,2368	0,2237
9,771428571	2	0,2857
8,408333333	2,7633	0,2303
8,484615385	3,0367	0,2336
9,822222222	2,5793	0,2866
.	1,3925	0,2785
9,55	3,3393	0,2783
7,925	1,7179	0,2454
8,8375	1,9911	0,2489
8,06	1,876	0,1876
.	0,831	0,277
10,76	1,5268	0,3054
11,76666667	1,7982	0,2997
6,18	2,5	0,1667
